# Cervical alignment and clinical outcome of anterior decompression with fusion vs. posterior decompression with fixation in kyphotic cervical spondylotic myelopathy

**DOI:** 10.3389/fnins.2022.1029327

**Published:** 2022-11-23

**Authors:** Wei Du, Hai-Xu Wang, Jing-Tao Zhang, Feng Wang, Xu Zhang, Yong Shen, Rong Chen, Li Zhang

**Affiliations:** ^1^Department of Orthopedics, The Third Hospital of Hebei Medical University, Shijiazhuang, China; ^2^Department of Neurology, Hebei Key Laboratory of Vascular Homeostasis, Hebei Collaborative Innovation Center for Cardio-Cerebrovascular Disease, The Second Hospital of Hebei Medical University, Shijiazhuang, China

**Keywords:** cervical spondylotic myelopathy, anterior decompression with fusion, posterior decompression with fixation, kyphotic correction, neurological improvement, axial symptoms

## Abstract

**Background context:**

Cervical kyphosis is a common but potentially debilitating and challenging condition. There is controversy on the optimal surgical strategy for the treatment of kyphotic cervical spondylotic myelopathy (KCSM) using either anterior approach or posterior approach.

**Introduction:**

The purpose of this study was to investigate the surgical efficacy of anterior decompression with fusion (ADF) vs. posterior decompression with fixation (PDF) for the treatment of KCSM, and to further analyze the changes of cervical spinal alignment parameters and axial symptoms (AS) severity after kyphotic correction.

**Materials and methods:**

We retrospectively reviewed 117 patients with KCSM who had undergone ADF (58 patients) and PDF (59 patients) between January 2016 and December 2020. Cervical spinal alignment parameters, including curvature index (CI) and C2-7 Cobb angle, were measured on the PreOP and PostOP lateral radiographs. Recovery rate was calculated based on the Japanese Orthopedic Association (JOA) score. AS severity was quantified by Neck Disability Index (NDI). A *P*-value less than 0.05 was considered to be significant.

**Results:**

The patient mean age, gender, presenting symptoms and follow-up time were similar between the two groups (*P* > 0.05). However, there were statistically significant differences (*P* < 0.001) between the two groups regarding the operation levels, operating time and intraoperative blood loss. Analysis of PostOP follow-up data showed significant differences (*P* < 0.001) in CI, correction of CI, C2-7 Cobb angle, and NDI between the two groups, whereas no significant differences in JOA score (*P* = 0.16) and recovery rate (*P* = 0.14). There were significant differences (*P* < 0.001) in CI, C2-7 Cobb angle, JOA score, and NDI between PreOP and PostOP follow-up in each group. Correction of CI showed positive correlation with recovery of NDI in Group ADF (*r* = 0.51, *P* < 0.001), and in Group PDF (*r* = 0.45, *P* < 0.001).

**Conclusion:**

Satisfied neurological improvement was obtained by ADF and PDF for patients with KCSM. Cervical kyphotic correction caused significant improvement of AS, and was more favorable with ADF than with PDF. Surgeons should pay full consideration of the merits and shortcomings of each approach when deciding on a surgical plan.

## Introduction

Cervical spondylotic myelopathy (CSM), which is a degenerative disease associated with cervical cord compression, usually results in a stepwise deterioration of neurological function and life quality. In some patients with CSM, cervical kyphosis may develop because of the changes of intervertebral height and sagittal lordotic alignment, as well as progressive degeneration of the discs and facet joints. Cervical kyphosis is a common but potentially debilitating condition that can be challenging to treat (Sivaganesan and Kim, 2022). Although non-surgical treatments may have some short-term benefits in improving this disease, surgery remained to be pivotal to decompression of the spinal cord and correction of kyphosis using either anterior approach or posterior approach.

In the past several decades, anterior decompression with fusion (ADF) has become the gold standard treatment for cervical degenerative disease related with radiculopathy and myelopathy (Chang et al., 2022). The anterior approach is particularly effective for directly decompressing the spinal cord, removing anterior bony spurs and disc fragments, restoring intervertebral height, and correcting segmental kyphosis. The ADF-related complications are not rare, such as iatrogenic spinal cord injury, dysphagia, hoarseness, and air-way obstruction, cerebrospinal fluid leakage, graft failure, and pseudarthrosis (McCormick et al., 2020; Lannon and Kachur, 2021). In recent years, posterior decompression with fixation (PDF) had been demonstrated effectively to provide better clinical outcomes than laminoplasty alone for CSM accompanying local kyphosis or segmental instability (Abumi, 2015). Some PDF-related complications, which include compressive epidural hematoma, vertebral artery injury, iatrogenic spinal cord injury, dural tears, and implant failure, have also been observed (Li et al., 2022). The optimal surgical strategy for kyphotic cervical spondylotic myelopathy (KCSM) remains controversial for spine surgeons.

The purpose of this retrospective study was to investigate the surgical efficacy of anterior ADF vs. PDF for the treatment of KCSM, and to further analyze the changes of cervical spinal alignment parameters and axial symptoms severity after correction of kyphosis.

## Materials and methods

### Participants

We retrospectively reviewed 139 patients who suffered from KCSM at our medical center from January 2016 to December 2020. Among them, 72 patients had undergone ADF; while others, 67 patients had undergone PDF.

The inclusion criteria include patients who had at least 1–5 levels of cervical spinal cord compression with combined symptoms and signs of CSM; and kyphosis defined as an alignment of C2-7 Cobb angle less than 0^°^ on lateral neutral radiograph. The exclusion criteria included (1) cervical trauma (*n* = 6 in Group ADF; *n* = 4 in Group PDF); (2) significant cervical anatomic deformity; (3) active infection; (4) rheumatoid arthritis; (5) neoplasm (*n* = 2 in Group ADF; *n* = 3 in Group PDF); (6) incomplete or poor quality pre- or post-operative magnetic resonance imaging (MRI) and X-rays (*n* = 4 in Group ADF; *n* = 1 in Group PDF); (7) loss to follow-up (*n* = 2 in Group ADF; *n* = 0 in Group PDF).

Finally, a total of 117 patients (58 patients in Group ADF; 59 patients in Group PDF) were included in this study. This study was approved by the Investigational Review Board at our institution, and informed consent was obtained from each patient.

### Surgical techniques

The patients in Group ADF underwent anterior cervical discectomy and fusion (ACDF) under general anesthesia. A standard right-sided approach through a transverse incision was used to expose the targeted segment based on the preoperative surgical planning. The compressive materials were removed, which included herniated disc, osteophytes and the posterior longitudinal ligament. The cartilaginous endplates were removed with a curette, and the bony endplates were protected simultaneously to prevent cage subsidence. The bilateral uncovertebral joints could be removed partially until release, but the vertebral artery must be taken care to avoid damage. The intervertebral space was properly distracted using a Caspar spreader, keeping the intervertebral space wide in anterior edge and narrow in posterior edge. An appropriate size Poly-ether-ether-ketone (PEEK) cage filled with excised osteophytes was implanted between vertebral bodies, and then the plate with normal cervical lordosis was fixed with screws inserted cranially and caudally.

Laminectomy with lateral mass screw fixation were performed in Group PDF by the same surgeon under general anesthesia. Screws (Medtronic Sofamor Danek, Memphis, TN, USA) were placed bilaterally with the Magerl technique (Pal et al., 2011), rods of appropriate size were selected and bent to match the normal cervical lordosis and secured to the lateral masses by screws, and then laminectomy were performed based on the preoperative surgical planning.

All patients stayed in bed for 1–3 days after surgery, and thereafter rehabilitation was instructed with a neck collar for 2 months. Anteroposterior, lateral, and flexion/extension lateral X-ray tests and MRI scans were routinely performed preoperatively. Routine X-ray tests were performed postoperatively at 3, 6, 12 months and then the last year.

### Radiological assessments

**FIGURE 1:**
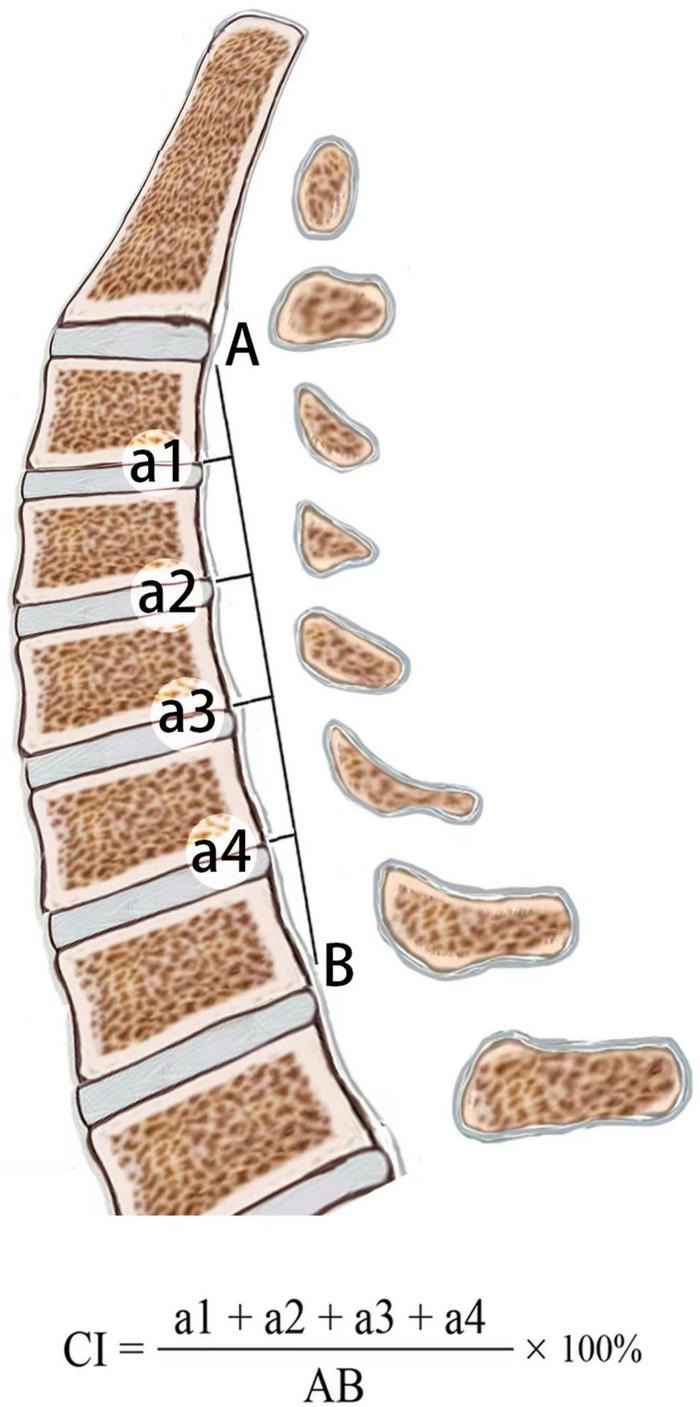
Calculation of the cervical curvature index (CI).

**FIGURE 2:**
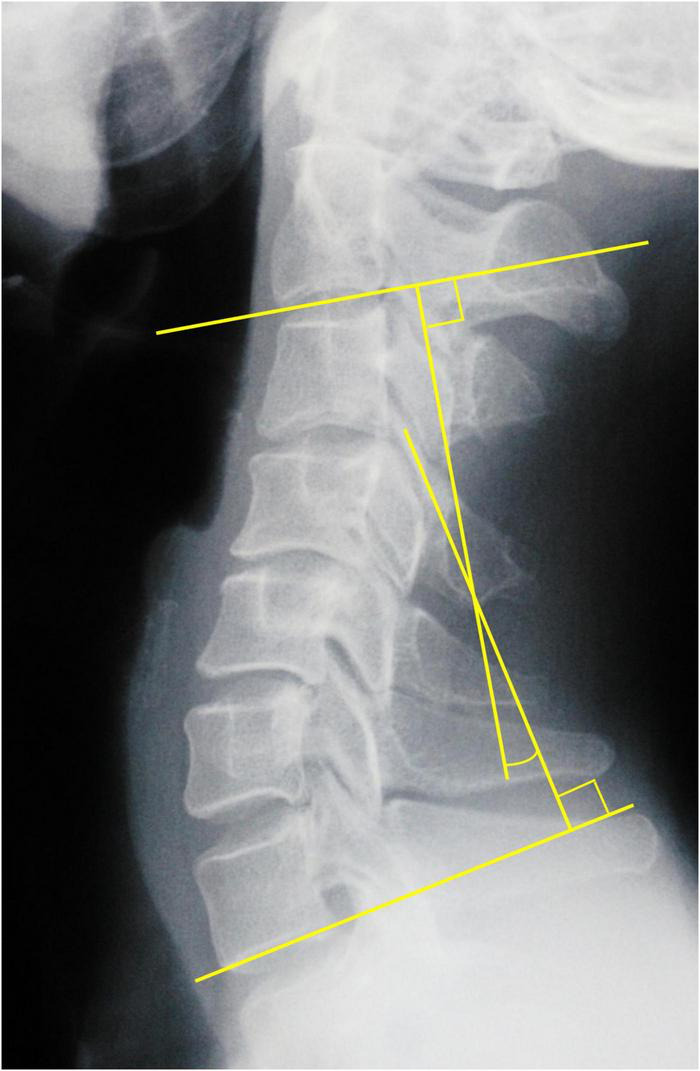
C2-7 Cobb angle measurement.

PreOP and PostOP follow-up cervical alignments were measured three times on standing lateral X-ray with 200% magnification for accuracy by the first and second authors independently, and the mean value was used for analysis. The intraobserver errors were less than 5%. Curvature index (CI, Figure 1): “a1” was defined as the distance from the posterior inferior edge of the C3 vertebral body to line “AB,” “a2, a3, and a4” using the same method; “AB” was defined as the distance from the posterior inferior edge of the C2 vertebral body to that of the C7 vertebral body. The correction of CI was calculated: PostOP CI–PreOP CI. C2-7 Cobb angle was the angle formed by the vertical lines of C2 and C7 inferior endplates in standing lateral radiographs (Figure 2).

### Clinical assessment

Patient’s neurological status was assessed using the Japanese Orthopedic Association (JOA) disability scale (Japanese Orthopedic Association, 1994). Neurological recovery rate was calculated using the Hirabayashi method: (PostOP JOA-PreOP JOA)/(17-PreOP JOA) × 100%. Recovery rates were graded as excellent (≥ 75%), good (50–74%), fair (25–49%), and poor (< 25%).

Axial symptom severity was quantified by Neck Disability Index (NDI, 0 = no disability, 50 = total disability) (Vernon and Mior, 1991). Subjects’ scores were calculated and ranked according to the NDI ranking system (no disability, 0–4; mild disability, 5–14; moderate disability, 15–24; severe disability, 25–34; complete disability, ≥ 35).

### Statistical analysis

All statistical analysis was performed using IBM SPSS Statistics, version 21.0 (IBM Corp., Armonk, NY, USA). Continuous variables were expressed as means ± standard deviation. The Chi-square test was applied for qualitative data. A paired *t*-test was used to assess statistical significance between PreOP and PostOP parameters in each group. Statistical comparisons between Group ADF and PDF were performed in the PostOP follow-up CI, C2-7 Cobb angle, JOA score, NDI, correction of CI, recovery rate and recovery of NDI using the independent sample *t*-test, and in the NDI ranking system using Mann–Whitney *U* test. The Chi-square test was performed in neurological recovery rate grade. Pearson’s correlation coefficient was used to check the correlation between correction of CI and recovery of NDI in each group. A value of *P* < 0.05 was considered to be statistically significant.

## Results

A total of 117 patients (58 patients in ADF group; 59 patients in PDF group) were included in this study (Table 1). The patient age, gender, presenting symptoms, and follow-up time were similar between the ADF and PDF groups (*P* > 0.05). However, there were statistically significant differences between the two groups regarding the operation levels (*U* = 883, *P* < 0.001), operating time (*t* = 12.13, *P* < 0.001), and intraoperative blood loss (*t* = 22.77, *P* < 0.001).

**TABLE 1:** Patient characteristics.

Characteristics	Group ADF	Group PDF
Total (*n* = 117)	58	59
Mean age (years)	60.47 ± 8.45 (45–74)	61.14 ± 7.17 (47–75)
**Gender**		
Male	42	39
Female	17	20
Operation levels*		
1 level	33	0
2 levels	21	1
3 levels	4	10
4 levels	0	31
5 levels	0	17
**Presenting symptoms**		
**Weakness**		
Upper extremity	45	47
Lower extremity	26	31
Extremity numbness hyperesthesia	34	35
Gait instability	38	40
Hyperreflexia	43	46
Hoffman sign	42	44
Babinski sign	21	25
Clonus	17	19
Operating time*	104.24 ± 24.96	151.86 ± 16.79
Intraoperative blood loss*	97.62 ± 29.72	357.80 ± 81.88
Follow-up time (year)	3.4 ± 0.9 (2–5)	3.6 ± 0.8 (2–5)

*Statistic tests: statistically significant differences between the two groups (*P* < 0.001).

### Radiographic results

**FIGURE 3:**
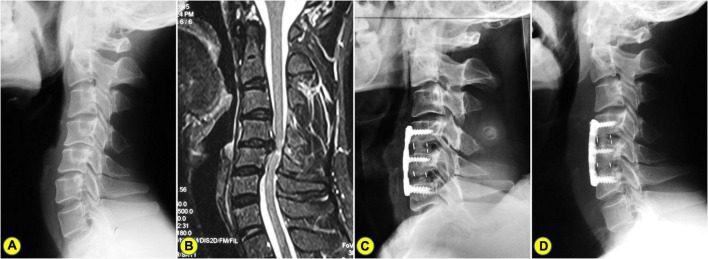
A 57-years-old man who underwent anterior cervical discectomy and fusion (ACDF). **(A)** The preoperative lateral X-ray shows the C2-7 Cobb angle and curvature index (CI) are 15^°^ and 9.49%, respectively. **(B)** Preoperative magnetic resonance imaging (MRI) showing C4-6 spinal cord compression combined with kyphosis. **(C)** A postoperative lateral X-ray showing ACDF. **(D)** On the lateral X-ray of 2.5 years after surgery, the C2-7 Cobb angle and CI were 7^°^ and 16.09%, respectively.

**FIGURE 4:**
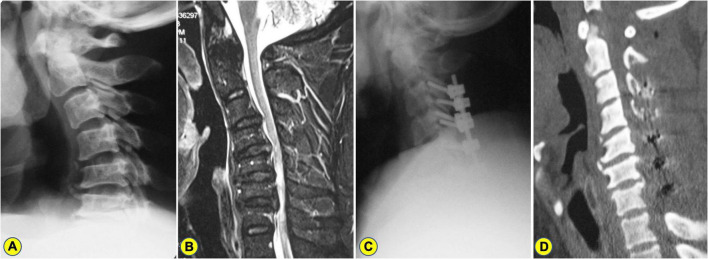
A 51-years-old man who underwent laminectomy with lateral mass screw fixation. **(A)** The preoperative lateral X-ray shows the C2-7 Cobb angle and curvature index (CI) are 17^°^ and 9.45%, respectively. **(B)** Preoperative magnetic resonance imaging (MRI) showing C3–7 spinal cord compression combined with kyphosis. **(C)** A postoperative lateral X-ray showing laminectomy with lateral mass screw fixation. **(D)** On the CT image of 3 years after surgery, the C2-7 Cobb angle and CI were 6^°^ and 13.09%, respectively.

**FIGURE 5:**
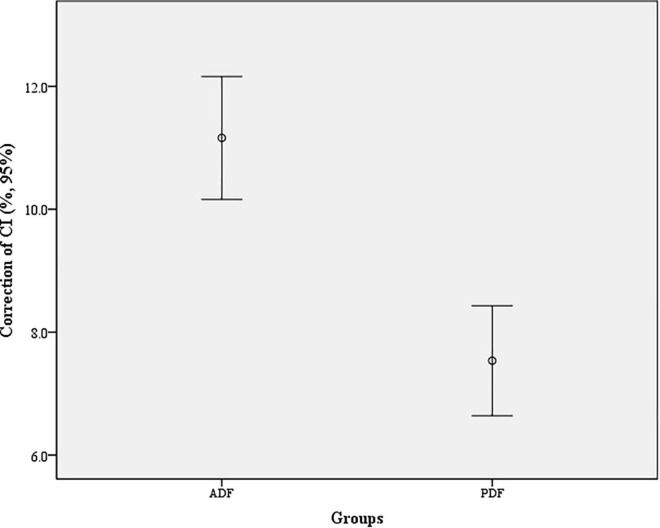
Correction of cervical curvature index (CI) in each group. The difference between the two groups for correction of CI was statistically significant (*t* = 5.41, *P* < 0.001).

In Group ADF, there were statistically significant differences between the PreOP and PostOP data regarding CI (*t* = 22.87, *P* < 0.001), C2-7 Cobb angle (*t* = 16.76, *P* < 0.001) (Figures 3A–D). In Group PDF, there were statistically significant differences between PreOP and PostOP data regarding CI (*t* = 16.95, *P* < 0.001), C2-7 Cobb angle (*t* = 13.09, *P* < 0.001) (Figures 4A–D). Between the two groups, there were no significant differences regarding the PreOP CI and C2-7 Cobb angle (*P* > 0.05). There were also statistically significant differences regarding the PostOP CI (*t* = 7.59, *P* < 0.001), correction of CI (*t* = 5.41, *P* < 0.001) (Figure 5), C2-7 Cobb angle (*t* = 6.39, *P* < 0.001) (Table 2).

**TABLE 2:** PreOP and PostOP follow-up cervical radiological data in each group.

Parameter	Group ADF (*n* = 58)	Group PDF (*n* = 59)	*t*-value*	*P-value*
**CI (%)**				
Preoperative	8.30 ± 2.36	8.17 ± 2.24	0.47	0.64
Postoperative follow-up	19.54 ± 2.87	15.71 ± 2.57	7.59	< 0.001
*t*-value*	22.87	16.95		
*P*-value	< 0.001	< 0.001		
Correction of CI (%)	11.16 ± 3.81	7.54 ± 3.44	5.41	< 0.001
**C2-7 Cobb angle**				
Preoperative	17.19 ± 4.63	16.69 ± 4.52	0.59	0.56
Postoperative follow-up	5.09 ± 2.97	8.15 ± 2.16	6.39	< 0.001
*t*-value*	16.76	13.09		
*P*-value*	< 0.001	< 0.001		

*t-test.

### Functional outcomes

There were statistically significant differences between PreOP and PostOP JOA scores in Group ADF (*t* = 26.09; *P* < 0.001) and in Group PDF (*t* = 26.31, *P* < 0.001), respectively. Between the two groups, there were no significant differences regarding PreOP and PostOP JOA scores (*t* = 0.37, *P* = 0.71; *t* = 1.40, *P* = 0.16) (Table 3).

**TABLE 3:** PreOP and PostOP follow-up Japanese Orthopedic Association (JOA) score and neurological recovery rate in each group.

Parameter	Group ADF (*n* = 58)	Group PDF (*n* = 59)	Statistic value	*P-value*
JOA score*				
Preoperation	8.78 ± 1.38	8.86 ± 1.21	0.37	0.71
Postoperative follow-up	14.97 ± 1.17	14.66 ± 1.18	1.40	0.16
*t*-value	26.09	26.31		
*P*-value	< 0.001	< 0.001		
Recovery rate (%)*	75.17 ± 14.33	71.20 ± 14.39	1.50	0.14
Neurological recovery rate grade**				
Excellent (≥ 75%)	31	22	4.13	0.25
Good (50–74%)	27	37		
Fair (25–49%)	0	0		
Poor (< 25%)	0	0		

*t-test, **Pearson Chi-square test.

The improvement rates were 75.17 ± 14.33% after ADF and 71.20 ± 14.39% after PDF, respectively. There was no significant difference between the two groups regarding the recovery rate (*t* = 1.50, *P* = 0.14). Based on the neurological recovery rate grade, the neurological recovery was excellent in 31 (53.4%) patients and good in 27 (46.6%) patients in Group ADF. In Group PDF, the neurological recovery was excellent in 22 (37.3%) patients and good in 37 (62.7%). According to Pearson Chi-square test, there was no significant difference between the two groups regarding axial symptoms (*χ^2^* = 4.13, *P* = 0.25).

### Axial symptoms

**FIGURE 6:**
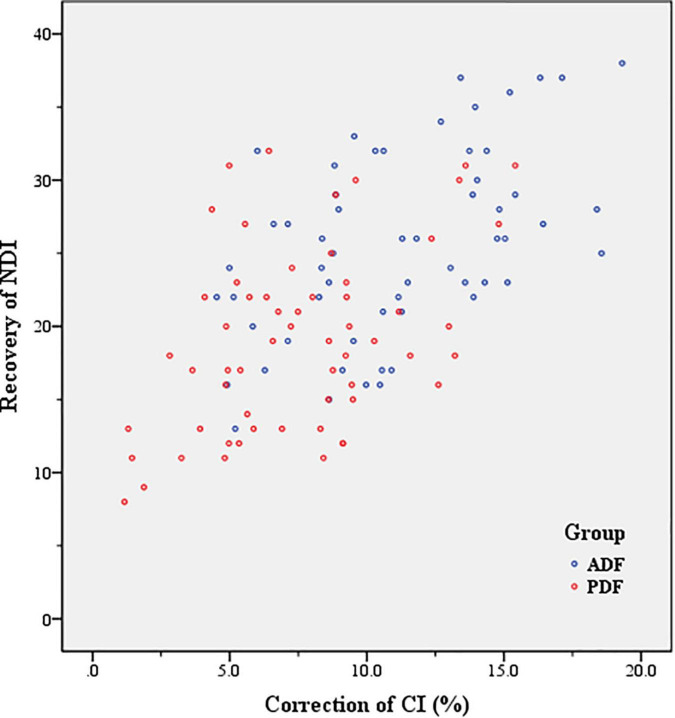
Correlation between correction of curvature index (CI) and recovery of Neck Disability Index (NDI) (Axial symptom severity) in Group anterior decompression with fusion (ADF) (*r* = 0.51, *P* < 0.001), and in Group posterior decompression with fixation (PDF) (*r* = 0.45, *P* < 0.001).

There were statistically significant differences between PreOP and PostOP NDI in Group ADF (*t* = 31.58; *P* < 0.001) and in Group PDF (*t* = 23.82, *P* < 0.001), respectively. Between the two groups, there was significant differences regarding PostOP NDI (*t* = 7.28, *P* < 0.001). The recovery of NDI was 25.53 ± 6.34 after ADF and 19.20 ± 6.31 after PDF, respectively. There was a significant difference between the two groups regarding recovery of NDI (*t* = 5.42, *P* < 0.001). There were positive correlations between the CI correction and recovery of NDI in Group ADF (*r* = 0.51, *P* < 0.001), and in Group PDF (*r* = 0.45, *P* < 0.001) (Figure 6 and Table 4).

**TABLE 4:** PreOP and PostOP follow-up Axial symptom severity [neck disability index (NDI) scores] in each group.

Axial symptoms	Group ADF (*n* = 58)	Group PDF (*n* = 59)	Statistic value	*P-value*
NDI*				
Preoperation	32.09 ± 4.33	31.51 ± 4.57	0.70	0.49
Postoperative follow-up	6.55 ± 4.37	12.31 ± 4.17	7.28	< 0.001
*t*-value	31.58	23.82		
*P*-value	< 0.001	< 0.001		
Recovery of NDI	25.53 ± 6.34	19.20 ± 6.31	5.42	< 0.001
NDI ranking system**				
No disability (0–4)	24	10	827.00	< 0.001
Mild disability (5–14)	33	32		
Moderate disability (15–24)	1	17		
Severe disability (25–34)	0	0		
Complete disability (≥ 35)	0	0		

*t-test, **Mann–Whitney *U* test.

Based on the NDI ranking system, there was no disability in 24 patients, mild disability in 33 patients, and moderate disability in 1 patient in Group ADF. In Group PDF, there were no disabilities in 10 patients, mild disability in 32 patients, and moderate disability in 17 patients (Table 4). According to the Mann–Whitney *U* test, there was a significant difference between the two groups regarding axial symptoms (*U* = 827, *P* < 0.001).

### Complications

Of a total of 58 patients in Group ADF, 14 patients (24.1%) suffered dysphagia, and 5 patients (8.6%) suffered hoarseness. However, dysphagia and hoarseness were relieved significantly after the above patients were given a low dose of glucocorticoid and dehydrant for 3 days. According to the recovery of NDI in Table 3, improvement of axial neck pain tended to be higher in Group ADF than in Group PDF, and the difference was significant (*t* = 5.42, *P* < 0.001). There were no major neurological or vascular complications, and wound complications in both groups.

## Discussion

Currently, there is still a significant debate on the optimal surgical strategy for the treatment of KCSM using either anterior approach or posterior approach. Our study found that the cervical kyphotic correction can improve the severity of axial symptoms (AS), and was more favorable with ADF than with PDF. This study also confirms that the correction of CI is positively correlated with the recovery of AS. Surgeons should pay full consideration of the merits and shortcomings of each approach when deciding on a surgical plan for the CSM patients with kyphosis.

### Options of surgical strategy for kyphotic cervical spondylotic myelopathy

Cervical kyphosis is a common but potentially debilitating and challenging condition. Surgical management for CSM patients with kyphosis aims to decompress the spinal cord and restore the normal sagittal alignment using either an anterior approach or a posterior approach. However, the above surgery-related complications include a high incidence of spinal cord injury, cerebrospinal fluid leakage, graft subsidence and extrusion, pseudarthrosis, fixation failures, and implant loosening (Ogura et al., 2021). Moreover, neurological recovery might be worsened because of the remaining anterior compression if the segmental instability and kyphotic deformity were not corrected effectively. The current evidence is not clear on whether anterior or posterior approach is superior for KCSM because of the shortcomings of each surgical approach.

Suda et al. (2003) thought that local kyphosis exceeding 13^°^ was a relative contraindication for posterior decompression and that then anterior decompression for correcting the kyphotic deformity should be recommended. Our early study (Du et al., 2014) showed that enlarged laminectomy with fixation (removing the inside edge of facet joints and decompressing the nerve foramina) was an effective strategy for improving neurological function, restoring the normal cervical lordosis, and decreasing the incidence of axial symptoms and C5 root palsy for multilevel cervical degenerative myelopathy (CDM) associated with kyphosis. In the present study, our results showed that both ADF and PDF produce similar neurological improvement, in agreement with the recent reports (Yoshii et al., 2020; Du et al., 2022). Therefore, we have good evidence that intraoperative adequate decompression of the spinal cord may be a pivotal factor in the early postoperative neurological recovery. Our results also showed a statistical difference between the two groups regarding the operation levels, and more operation levels were performed using PDF compared to ADF. Surgeons should pay full consideration of the merits and shortcomings of each approach when deciding on a surgical plan. ADF is more suitable for the KCSM patients who have less than 3 levels of spinal cord compression which need to be intervened. Nevertheless, PDF is more suitable for the KCSM patients who have 3 or more levels of spinal cord decompression.

### Intraoperative consideration of anterior decompression with fusion vs. posterior decompression with fixation for kyphotic cervical spondylotic myelopathy

Surgery-related complications are relatively rare and mainly included compressive epidural hematoma, vertebral artery injury, dural tear, and iatrogenic spinal cord injury. Whether anterior or posterior approach is performed, the key point for the surgeon is spinal cord decompression with special attention to avoid iatrogenic spinal cord injury. Intraoperative neuromonitoring (IONM), including motor evoked potentials (MEPs), somatosensory evoked potentials (SSEPs), and electromyography (EMG), had gained popularity for the potential detection of neurological injury during spinal surgery (Mesregah et al., 2020). IONM afforded the surgical team an opportunity to perform rapid intervention and prevent injury progression or possibly to reverse impending neurological sequelae.

To avoid large amounts of bleeding and postoperative epidural hematoma, the surgeon must pay careful attention to hemostasis. For example, bipolar electrocautery and absorbable gelatin sponge were used for hemostasis in the epidural space, and bone edges were waxed as necessary. Tranexamic acid (TXA) can significantly reduce perioperative blood loss in cervical, thoracic, and lumbar laminectomy and fusion procedures, while demonstrating a minimal complication profile (Brown et al., 2022).

Lateral mass screw fixation is commonly used in the fusion and stabilization of the subaxial cervical spine. The accuracy of screw trajectory, screw length, technique of insertion, vertebral level, and size of the lateral mass affect the safety of lateral mass screw placement (Evans et al., 2022; Soliman et al., 2022). The main complications were lateral mass fracture and instrumental failure, resulting in screw breach into the ventral soft tissues, which may injure vertebral artery, roots and cervical sympathetic ganglion. With the progressive degeneration of facet joints, cervical lateral mass morphology may change. It is strongly necessary to assess the preoperative anatomical changes of cervical kyphosis using a 3D CT scan. For patients with severe cervical anatomical deformity, intraoperative CT navigation can enhance the safety of surgery (Sabri and York, 2021). Successful lateral mass screw placement still highly depends on the surgeon’s experience. In the present study, there were no patients who developed the above surgery-related complications.

### Correction of cervical kyphosis *via* anterior decompression with fusion vs. posterior decompression with fixation

Wu et al. (2021) found that cervical focal kyphosis correlated with worse myelopathy symptoms, and the threshold cervical focal kyphosis for severe myelopathy symptoms is predicted to be at about 7^°^. Progressive cervical kyphosis may result in cervical pain, which seriously decreases patients’ quality of life. When the patient is supine during operation, cervical spine is elevated as high as possible to restore the cervical lordosis. Intraoperative cervical anterior or posterior fixation should keep to normal cervical physiologic lordosis. In the current study, cervical kyphosis can be corrected effectively by both ADF and PDF, and was more favorable with ADF than with PDF. We speculate that the cervical anterior column is more advantageous than the posterior column in the kyphotic correction.

### Complications of anterior decompression with fusion vs. posterior decompression with fixation for kyphotic cervical spondylotic myelopathy

Dysphagia and hoarseness are the most common complications after ADF, with incidences ranging from 2.7–33.3% and 3–11%, respectively (Bakhsheshian et al., 2017; Nagoshi et al., 2017). The possible causes include postoperative soft tissue edema, postoperative hematoma, esophageal injury, cervical plate implanting, and the surrounding scar formation (Shen et al., 2018; Tetreault et al., 2022). In the current study, these complications usually occur in patients with multilevel spinal cord compression, longer operation times and short wide necks. However, dysphagia and hoarseness were relieved significantly after the patients were given a low dose of glucocorticoid and dehydrant.

The incidence of axial symptoms can be up to 29.91% after ACDF (Zhou et al., 2018) and 19.25% after laminoplasty (Chen et al., 2021). Postoperative neck pain alleviation may be related to the transient relief of facet joint pressure during the vertebral distraction procedure in ACDF (Xu et al., 2022). In the present study, cervical kyphotic correction causes significant improvement of axial symptoms postoperatively than preoperatively. The results show that improvement of axial symptoms is positively correlated with cervical kyphotic correction, and is more favorable with ADF than with PDF. Posterior decompression of spinal cord bases on disruption of the posterior tension band while cervical kyphosis is corrected. However, anterior decompression of spinal cord, as well as kyphosis correction, do not involve in disruption of the posterior tension band. We speculate that cervical kyphotic correction can change the bio-mechanical distribution of discs and facet joints, further resulting in postoperative neck pain alleviation.

### Limitations of present study

This study has limitations. First, we only included the patients with KCSM. Second, all patients were selected from a single hospital and all operations were performed by the same surgical team, which may produce a selection bias. Third, only ACDF was selected for anterior approach, and only laminectomy with lateral mass screw fixation was selected for posterior approach. Fourth, our retrospective design has inherent weaknesses that may produce a statistical bias. Fifth, multi-center long-term clinical trials should be performed to ascertain the results of this study.

## Conclusion

Satisfied neurological improvement was obtained by ADF and PDF for patients with KCSM. Cervical kyphotic correction caused significant improvement of AS, and was more favorable with ADF than with PDF. Surgeons should pay full consideration of the merits and shortcomings of each approach when deciding on a surgical plan. ADF is more suitable for the KCSM patients who have less than 3 levels of spinal cord compression which need to be intervened. Nevertheless, PDF is more suitable for the KCSM patients who have 3 or more levels of spinal cord decompression.

## Data availability statement

The original contributions presented in this study are included in the article/supplementary material, further inquiries can be directed to the corresponding authors.

## Ethics statement

The studies involving human participants were reviewed and approved by the Ethics Committee of The Third Hospital of Hebei Medical University. Written informed consent for participation was not required for this study in accordance with national legislation and institutional requirements.

## Author contributions

WD participated in the study design, collection, interpretation, and writing of the manuscript. H-XW, J-TZ, and FW participated in the collection, interpretation, and analysis of the data. XZ and YS participated in revising of the manuscript. RC and LZ participated in the study design. All authors contributed to the article and approved the submitted version.
